# The Report of a Committee on the New Poor Law Act, Appointed by the Provincial Medical and Surgical Association, at the Anniversary Meeting Held at Oxford, and Read at the Anniversary Meeting, Held at Manchester, July 21, 1836

**Published:** 1836-10-01

**Authors:** 


The Report of a Committee on the New Poor-Law Act, appointed by the Provincial Medical and Surgical Association, at the Anniversary Meeting, held at Oxford, and read at the Anniversary Meeting, held at Manchester, July 21, 1836. Octavo, stitched, pp. 38. Worcester, 1836.

This Report is on a subject of great importance to the bulk of medical practitioners in England. It has come to hand so late, that we can do little more at present than offer an exposition of the main facts that it contains, and of the remedies for alleged grievances that it proposes.

There can be no question that a crisis had arrived in the condition of English paupers, when the laws relating to them came under revision. The old laws had made the poor paupers—had degraded and demoralized the paupers that it made—and was exerting the same destructive influence on the classes of society above the poor. If ever there were laws calculated for the misery of a population, such were the old poor-laws. Bad in principle, worse in practice, they were spreading idleness, vice, and destitution through the land, and—

"The bold peasantry, their country's pride,"

were sinking fast to the very lowest abyss of wretchedness and profligacy. Had such a state of things continued for another century, a servile war must have resulted.

The institution of the Poor-law Commission, and the Poor-law Amendment Act, based on its Reports, achieved such a complete and sudden revolution in the condition of the class that it referred to, as is probably unequalled in legislative history.

The principle of the New Poor Law is a simple one. It says that the condition of the pauper must not be made an enviable one. It must not be a prize for the idle and the vicious. A very proper principle, a principle consistent with reason, and with the practice of every individual in private ife. But that which was so rational, and which men always acted on in the management of their own families, indolence and a false philanthropy led them to neglect in the regulation of the pauper class.

As a corollary from this principle it follows, that whilst the pauper is an object of state relief, the merely poor are not. If the poor man knows that his income will be eked out without increased exertions of his own, he has little inducement for industry. Additional exertion can only procure him that which he gets without any exertion at all. "Why should I work," said the labourer, "when I can live without it?" He did not work, man never will work, when idleness will procure him a subsistence. If this were ever doubted, the history of the English poor has proved it.

The principle of the old law was to assist the *poor* man—the principle of the new law is to refuse to assist him. The old law is said to be humane. See what it did. A penny-wise and pound-foolish humanity, which gives one meal and impoverishes an existence. Yet it is for the re-establishment of such a system so vicious in principle, so corrupt in practice, so mischievous in results, that the small fry of legislators, and the

Petits amis de droits de l'homme, are clamouring. These people are incorrigible. Reasoning—facts are thrown away upon them. They turn from a mass of authentic evidence to the pot-house speeches of disappointed philanthropists, whose grumbling is essentially grounded on their inability to encourage their benevolence as heretofore, with dinners of white-bait at the public charge.

The evidence already collected of the working of the New Poor-law Act, has shewn its beneficial influence on the poor themselves. Here and there, no doubt, hardships have occurred, and in all great changes of system must occur. The wonder is, that they are not greater. In spite of them, the fact of the progressive improvement of the pauper population remains uncontradicted by any thing like satisfactory evidence, and whilst the rate-payers are relieved, the *rate-paid* have been benefited. The essential benefit, however, is to come—is to be looked for some years hence in the condition of those who are now the children of the paupers. Habits of industry and of dependence on their own exertions are generated, and those who are really interested for the well-being of fellow-creatures, have grounds for satisfaction in the prospect of the future.*

But it may be questioned whether the general principle on which the New Poor-law is administered is applicable to the *sick* pauper—whether medical attendance should not be furnished on a more liberal scale than other necessaries.

That the pauper should have medical advice is clear. We give it to the felon. The only difficulty in this case is to combine justice to the rate-payer with justice to the pauper, and justice to the medical man with both. The difficulty of the case is extreme, and it is set forth in strong colours in the present pamphlet.

Should medical advice be given by the State to the mere poor? We think there can be little question that, with certain limitations, it should. The poor man cannot be expected to do much more than support himself with necessaries. Medical advice is always expensive, for the mere cost of medicines is great. It is a luxury beyond his reach ; and the more so, because it is usually wanted in proportion as the ability of labour diminishes. In a labourer's family, if the labourer himself is sick, the whole income of the family is stopped, and at the same time an additional expense is incurred. But whilst we grant medical relief to the poor, we should encourage them, gently encourage them, to do their best, by benefit societies and associations of that description, to procure medical assistance for themselves. Judiciously constructed benefit societies are still a desideratum for the lower class of artisans and labourers; and there is no doubt of their more general adoption, as this class is thrown on its own resources.

For the present, we shall say nothing on self-supporting dispensaries, but proceed to examine the manner in which the Poor-law Commissioners have acted towards the medical profession. The best way, on this occasion, is simply to set forth the facts that the Committee of the Provincial Association have elicited. Those facts should be widely known, in order that there may be data for the discussions which are impending in Parliament, and are actively going on out of it.

The Committee observe, that the bulk of medical men have evinced a backwardness in forwarding statements of the circumstances which have occurred within their knowledge. The reasons are obvious—indolence in some—acquiescence in favourable arrangements in others—the terror of local influence in the rest.

The Committee start with claiming for the profession a due remuneration for attendance on the pauper. The medical man contributes his share to

* We would advise our medical brethren to disregard the general declamation on this subject, and to read the Reports of the Commissioners, and the articles on the subject which have appeared in the Edinburgh Review.

the support of the pauper. He cannot be also called on to contribute his time, his talents, and, in short, his capital, to the pauper when afflicted with disease. This would be to doubly tax him.

It is equally clear, that if medical advice is granted to the pauper at all, it should be good medical advice. The most cold-blooded inhumanity could not defend the principle of giving him advice *known* to be bad. The Committee, indeed, form a high estimate of the qualifications of a parish surgeon.

"The office of parish surgeon should combine the highest qualifications of the medical body: it being clear that no professional responsibility whatever, public or private, equals it in variety and extent. It embraces and presents, in daily profusion, cases in medicine, surgery, and midwifery, requiring as profound knowledge and diligent care as any public Institution affords for each of these branches singly. It should unite, therefore, an acute perception of the incipient stages of disease, with well-directed efforts for its prevention, accompanied by the most diligent and scientific treatment." 8.

The combined talents of a Brodie, a Baillie, and a William Hunter, would thus appear to be necessary for the proper performance of the functions of a parish surgeon. Unfortunately, too many have not been sensible of this responsibility, for it is notorious that much of the attendance on the paupers has been delegated to assistants and apprentices.

It appears that the Committee have directly or indirectly received accounts from forty-eight individuals, respecting the arrangements in forty-seven unions. The Committee class the results of these communications under three heads.

1. *Remuneration of the Medical Officer.*

"The practice of requiring 'Tenders' has evidently become more general than formerly. It appears to your Committee unnecessary to dwell upon the evils of this absurd and pernicious custom; they have already been sufficiently exposed: the feelings and opinions of every member of our profession must surely be unanimous on this particular. But the point of view in which, at the present time, it is desirable that the 'Tender' system should be placed, is the *degradation* that it inflicts on professional men. In many unions, however, instead of 'Tenders' being required, and also in those where 'Tenders' have not been found to reduce the medical stipend so low as was thought proper, the Guardians have fixed a sum for medical services, subject to the approbation of the Commissioners. This, although apparently different from 'Tenders,' amounts, in reality, nearly to the same thing; for the parties who fix the remuneration, are not guided in their determination by a careful estimate of the intrinsic value, or of the ordinary price of medical attendance; but merely by the circumstance that persons *can be found* who will undertake the duties on such terms; the offers of the Guardians are, therefore equivalent to the 'Tenders' of an adventurer, and have the same effect." 12.

It appears that a year's experience has shewn that, in the greater number of instances, the remuneration has not been equal to that obtained from the old mode of contract, and has proved grossly insufficient. This has been more particularly the case in the smaller parishes, and in those distant from medical advice.

The mode of remuneration thus set forth being, perhaps, too high, the advantages of the medical officer too great, a new method has been lately adopted, and a species of medical club has been established, for the purpose of providing medical relief, both for the independent labourer and the pauper. The following is a specimen of the manner in which these medical clubs are to be constituted. We introduce a rescript of the Epping Board of Guardians, confirmed by the Assistant Commissioner.

"The remuneration to the medical officers shall be by annual subscriptions of the Guardians, and of such of the independent labouring poor as may be desirous of availing themselves of the proposed arrangement within the prescribed period, according to the following rates:—

| | *s.* | *d.* |
|---|---|---|
| For an individual maintaining himself or herself .................. | 2 | 6 |
| For a wife, whose husband is a member of some benefit society.... | 2 | 0 |
| For a man and his wife ........................................ | 4 | 0 |
| For each child in a family (if one be subscribed for, all must)...... | 0 | 6 |
| For every person in the same family .... .. .................. | 2 | 0 |
| Cases of midwifery ........................................ | 10 | 0 |

The Guardians are to have the privilege of adding to the pauper schedule any name they may think proper, during the contract, paying only at the same rate as for those originally included." 15.

It is obvious that the power reserved to itself by the Board of Guardians is open to the strongest objections. The very principle of a "medical club" is, that the healthy pay for the sick. It is clear that, if every individual who pays half-a-crown a-year were sick, the doctor must speedily be ruined. The Board of Guardians, we may be certain, will only add the sick, and not the well, to the schedule. Take the following illustration of what may be done in this way.

"In the parish of Chigwell, the pauper population is estimated at four hundred, *i. e.* those who when ill would receive parochial relief. Out of this number only *one hundred* are contracted for, as receiving weekly allowance from the parish; so that on any of the others requiring medical aid, they are immediately placed on the 'schedule,' thus rendering the parish surgeon liable to attend *all*, without *all* being *contracted for*." 16.

Well may the Committee say, that one general feature pervades the several modes of remuneration—they are all utterly inadequate to recompense the medical officer.

### 2. *Medical Appointments and Duties.*

"The diminution in the number of medical attendants on the poor, is one of the principal features in the present system. It may be said, that as there are fewer paupers to be attended, it is reasonable that fewer medical men should be employed. This argument might apply with some force, if the paupers were more closely congregated, but the case is very different. The paupers inhabit the same extent of country as before, and therefore, in rural districts, the labour of the attendant is scarcely at all diminished, on account of the diminution in the number of his patients, while, for other reasons hereafter to be mentioned, his trouble is much increased.

Your Committee are in possession of numerous facts bearing on this point.

In the Cookham and Bray unions, *two* medical men were appointed under the new system, in place of *seven* who previously attended.—In the Newbury union, consisting of *eighteen* parishes, *one* individual undertook the duties formerly performed by *twelve;* he had no assistant, and had a space to ride over measuring sixteen miles by ten.—In the Bampton district of the Witney union, *ten* miles in diameter, *eight* medical men were formerly employed, now only *one*.—In one of the districts of the Aylesbury union, the surgeon resides at a distance of *seven* miles from one part of the district, where medical assistance might be obtained within *two* miles.—In another district of the same union, the nearest point to the surgeon's residence is *seven* miles, and the most remote *twelve*. Under the old system *sixteen* medical men were employed for the parishes of this union, containing forty parishes and four districts; under the new system *three* medical men only, one of whom was likewise appointed to an extensive district in another union.—In the Wheatenhurst union, comprehending *fourteen* parishes, and necessarily much travelling, the Commissioners induced the Guardians to waive a contract with the established practitioners, and to engage *one* young man from the schools, who had neither a horse nor instruments.—In the Faversham union, including twenty-five parishes, only *one* medical man was employed.—Numerous cases of a similar kind could be adduced." 17.

The districts divided and devised for general purposes have been made medical districts without reference to the convenience of procuring medical assistance. This is particularly exemplified in the Thame union. The distribution of the parishes in the districts of this union is so unsuited for medical purposes, that the nominal district surgeons were unable, by themselves, to perform the duties required. In the Ivor district of the Eton union, formerly *four* established medical men were located near the boundary, at three different convenient stations: under the present arrangement, *one* only has the charge, and he residing at an extreme corner of the district. It certainly does appear most unjust to the patient, to appoint him a surgeon residing many miles off, when one could be found much nearer—and most annoying to the surgeon to require that, for a scanty remuneration, he should be put to such extreme and unnecessary inconvenience. The Committee remark that a singular practice has crept in, to obviate the difficulties which have thus arisen. The nominal medical officer of the district has been permitted to engage other practitioners, residing in different parts of his district, making his own terms with them. This, be it observed, has been entirely *optional* with the medical officer: the Guardians merely stating that they should consider him *responsible* for the whole.

Responsible! This is laughable enough. Surely the Guardians could not keep their own countenances, when they uttered that melancholy joke. But this is not all.

"The proper performance of medical duties has, moreover, received another check by the new regulations. The power of deciding as to the necessity of medical aid in any case of illness, has been vested in the relieving or parish officer. Hence a fair opportunity for the early and successful treatment of disease, has often been lost to the medical man, and his judgment fettered by his necessary dependance on the discretion of these inferior officers." 19.

To be sure there is here a choice of difficulties. On the one hand there is a malingering pauper, and a medical man whose interest it is, *unless* there was a contract, that the pauper should be sick. We do not say that such a feeling would influence the majority of medical men. But, unfortunately, necessity operates too powerfully on many of our profession both in town and country, and however unwillingly we may open our eyes to the possibility of occurrences of this sort, those out of the profession will and do make it an element in their calculations. But if there is a chance of evil in allowing the medical man to decide, there is a still greater chance of a person ignorant of medical matters, *and* whose interest it is that the pauper should not be sick, having to determine the point in question. Such an arbiter will be very likely to refuse relief to persons in the early stages of disorders. When we consider the distant residence of the relieving officer, his constant occupation, and the parish officers' frequent unwillingness to act, added to the increased remoteness of medical advice, we may well believe that dangerous delay has occurred, and, under the present system, will still occur.

3. *Liability of the Medical Officers to Insult.*

The evidence collected by the Committee goes to shew that medical men in general, and especially those engaged in parish practice, are liable to offensive and degrading treatment from the new authorities. They are summoned and may be reprimanded by the Board of Guardians, and are by no means treated with the consideration due to a scientific and benevolent profession.

Such are the complaints against the operation of the New Poor-law on the profession. They may be resolved into imperfect remuneration, and vexatious or, at all events, inconsiderate regulations. It may be urged:—" If the remuneration is really imperfect, if no indirect advantages compensate for the scanty payments from the pauper-fund, why do not medical men refuse to do the work, why do they struggle in a ruinous competition with each other." Such a question would be reasonable, and the reply to it exhibits in the strongest manner the necessity that exists, not only for professional union, but for some controlling power which shall check those slighter professional delinquencies not cognizable by the existing tribunals.

The reason, then, why medical men have been forced to accept the proposed scale of remuneration on ruinous terms, is this:—The Commissioners invite young men just embarking in practice, and allure them with the *spolia opima*, not of the parish, but of the surrounding country. They know that young men, to get a footing in a place, will gladly take the parish, or the union, for the very lowest possible remuneration. Parish practice introduces to other practice, and an adroit well-informed young man, who has the parish, may do serious damage even to the established practitioner who has it not. This is the rod that the Commissioners keep in pickle for the refractory, and a very effectual rod it has been. But while we give the Commissioners credit for the cleverness, we cannot compliment them on the honourable or proper feeling of the manœuvre. It may be effectual, in a great degree it has been so, but if it rouses an influential profession to hostility against the Poor-law System, its ultimate policy may be questioned. The tact of the Commissioners has been considerable, and their inquiries into the abuses of the old poor-law have taught them not a little astuteness. Let a thief catch a thief, is a motto that they appear to have got by heart and to have acted on.

They have plausibly argued that the competition is fair and open. They know pretty well the sort of fairness it embodies.

We have gone through the principal facts of general importance, contained in this Report. They prove two things: first, that the Poor-law authorities naturally endeavour to drive the hardest bargain with the medical profession; and, secondly, that the profession is not true to itself.

The Poor-law authorities are peculiarly circumstanced. A furious and insane clamour has been incessantly directed against the New Poor-law. A

part of the press lives by pandering to the praiseworthy but ignorant benevolence of the mob of every class. To meet this organized and formidable opposition, the Poor-Law Commissioners have been obliged to enlist some strong feeling of the community on their side. They *have* enlisted a very strong one, its avarice. The old poor-law was extravagant—this is economical. The saving already effected is immense. That circumstance alone will defeat the opponents of the measure.

The pressure from without has contributed, no doubt, to increase the disposition to clip the " profits" of the medical practitioner to the uttermost. But it is notorious that the ultra-economists have not hesitated to denounce gratuitous medical assistance. Lord Brougham is reported to have denied the utility of Hospitals and Dispensaries.

These considerations may explain, but they do not justify the conduct of the Commissioners. These gentlemen have evinced, so far as we can see, no especial coyness in accepting salaries themselves. They have not been public-spirited enough to put up their own places to the chances of a " tender." We may allow something, no doubt, for their consciousness of the possession of supreme ability. Yet, withal, they contrive to fatten on the poor. What *right*—what right, we mean, in equity, have these well-paid functionaries, who draw their thousands annually from the savings of the poors'-rates, to demand that medical men should expend their time, and occupy their skill for nothing. No right save the tyrant's—power. Justice and reason are against them—their own well-lined paunches cry fie on them—and they may be sure that what is inconsistent with fairness will not, in the long run, be beneficial to the best cause. Our remarks on this head are impartial, because we neither are, nor by possibility can be, parish-surgeons—and that they are unprejudiced will probably be conceded, as we do not belong to the thick-and-thin opponents of the New Poor Law. This we think, on the whole, to be a wise, and, in its final effects, a humane enactment. But we do not think that the proceedings of the Commissioners, in respect to medical relief, are characterized either by wisdom or humanity.

The true remedy is in the hands of the profession itself. One thing alone is wanted, to secure it victory in a just demand for *moderate* remuneration for its services. That one thing is, however, hard to get—it is *union.* We want some central power, which can morally and effectually punish the crime of lese-majesté against the honour, interests, or dignity of the profession. We want some body similar to the Law Association. We are not without hopes that, sooner or later, we shall have it. Our brethren in the country have been industriously prejudiced against the medical men of the metropolis. The general practitioners have been arrayed against the physicians and surgeons, and, in return, the latter have learnt to regard the former with jealousy. How foolish, how injurious this is, the present proceedings are determining. On this schism the enemies (no, we will not say the enemies, but undoubtedly *not* the friends) of the profession reckon; and they bully the respectable surgeon in the country with the protégé of some London teacher.

The Provincial Association is, we trust, the nucleus of one which will not be provincial—of one which will embrace the members both of town and country. That, however, is not yet the case. The shoe pinches now, and it must be eased at once. Our advice to our country friends is this. Continue to collect facts—make on every occasion suitable, but gentlemanly remonstrances to the Boards of Guardians and Commissioners—put the public in possession of the case—urge it with reasoning and with absolute truth, not with clamour and exaggeration—and appeal on all suitable occasions to the Legislature. Above all things be united, but united as a scientific profession should be, not as a set of political brawlers. You may be assured that, as in all other cases, justice will prevail in the long run, and that reason will find its way to the interior of the most bigoted bosoms.

We say nothing of the recommendations of the Committee, partly because that would be unseasonable, and partly because we do not altogether approve of them.

